# Red Clover Isoflavones Influence Estradiol Concentration, Exercise Performance, and Gut Microbiota in Female Mice

**DOI:** 10.3389/fnut.2021.623698

**Published:** 2021-04-14

**Authors:** Yi-Ming Chen, I-Lin Wang, Xin-Yi Zhu, Wan-Chun Chiu, Yen-Shuo Chiu

**Affiliations:** ^1^The College of Physical Education, Hubei Normal University, Huangshi, China; ^2^Graduate Institute, Jilin Sport University, Changchun, China; ^3^School of Nutrition and Health Sciences, College of Nutrition, Taipei Medical University, Taipei, Taiwan; ^4^Research Center of Geriatric Nutrition, College of Nutrition, Taipei Medical University, Taipei, Taiwan; ^5^Department of Orthopedics, Shuang Ho Hospital, Taipei Medical University, Taipei, Taiwan

**Keywords:** red clover, estrogen, isoflavone, gut microbiota composition, intestinal health

## Abstract

In red clover (*Trifolium pratense* L.; RC) the main compound is isoflavones, which are selective estrogen receptor modulators for maintaining female health. Isoflavones exert antifatigue effects during exercise in high-temperature environments. This study aimed to investigate the effect of RC supplementation on gut microbiota composition to determine whether it improves intestinal barrier function and exercise performance. Female ICR mice were divided into four groups (*n* = 8 per group) and orally administered RC once daily for 6 weeks at 0 (vehicle), 308 (RC-1X), 615 (RC-2X), and 1,538 (RC-5X) mg/kg. RC supplementation decreased the fat mass and increased exhaustive swimming time, grip strength, and muscle glycogen in female mice. In the RC supplementation group, serum levels of lactate, ammonia, and creatine kinase decreased after swimming. The estradiol and progesterone levels were higher in the RC group than in the vehicle group. Regarding gut microbiota composition, the RC-2X group may increase intestinal health related to the microorganisms *Pseudobutyrivibrio* and *Parabacteroide*. Thus, the use of RC supplements as nutraceuticals could have positive effects on athletes' gut and overall health.

## Introduction

Red clover (*Trifolium pratense* L.; RC) is one of the most important leguminous plants of temperate zones in Europe and Asia, and it is also a short-term perennial herb (1, 2). RC contains high levels of isoflavones (genistein, daidzein, biochanin A, and formononetin) (3). With the discovery of the medicinal value of RC, many studies have shown that the chemical properties of isoflavones extracted from RC are similar to those of estrogen and can thus regulate estrogen levels in the human body. RC is one of the major sources of isoflavones (4, 5). In recent years, RC has been used in ameliorating menopausal symptoms, such as hot flush, night sweats, urogenital atrophy, depression, and anxiety, in postmenopausal women (6). In addition, RC can contribute to postprandial blood glucose level reduction, improves hypoglycemia, is an antioxidant, and has effects against obesity in a rat model (7) and osteoporosis in postmenopausal women (3, 7–10).

Estrogen plays physiological roles in reproductive organs and nonreproductive organs/tissues (11). Nagai et al. (11) showed that estrogen is a sex steroid hormone beneficial for exercise endurance and energy expenditure in female mice. Studies have shown that estrogen can promote the expression of myogenic differentiation 1 mRNA and muscle protein synthesis in postmenopausal women (12). It has a positive effect on the growth and regeneration of skeletal muscles and can promote recovery after sports injury (13, 14). Furthermore, estrogen is beneficial in maintaining muscle strength (15). Estrogen therapy or phytoestrogen supplementation may enhance the effects of exercise on bones and muscles mass (16).

Some studies have shown that strenuous exercise can reduce blood flow to the intestinal wall (17–19). Endurance athletes in particular are more likely to develop gastrointestinal diseases (20). Supplementation of isoflavones through a fermented red clover extract reduces markers of protein degradation (21), increases lean body mass and muscle mass in postmenopausal women (22).

Thus, we used RC, which has detectable estrogen activity and contains a high amount relative effect on estradiol (E2), as a supplement to determine the relationship between exercise performance and the gut microbiota. This study demonstrated that RC improved exercise performance and did not harm the intestinal health of female mice.

## Materials and Methods

### Animals and Experiment Design

The RC extract (T. pratense) was purchased from NOW FOOD (IL, USA). Female ICR mice (6 weeks old) grown under specific pathogen-free conditions were purchased from Liaoning Changsheng Biotechnology (Shenyang City, China). The animal protocol (IACUC-19003) was reviewed and approved by the Institutional Animal Care and Use Committee (IACUC) of Jilin Sport University, Changchun City, China. All mice were provided a standard laboratory diet (No. 5001; PMI Nutrition International, Brentwood, MO, USA) and distilled water *ad libitum*. Housing conditions included a 12-h light-dark cycle and air maintained at room temperature (22°C ± 1°C) with 30–40% humidity. The 1X dose of RC used for humans is typically 1,500 mg per day. The 1X mouse dose (308 mg/kg) we used was converted from a human-equivalent dose (HED) based on body surface area according to the US Food and Drug Administration formula: Assuming a human weight of 60 kg, the HED is 1,500 (mg)/60 (kg) = 25 × 12.3 = 308 mg/kg; the conversion coefficient 12.3 is used to account for differences in body surface area between mice and humans, as previously described. In total, 32 mice were randomly assigned to four groups (eight mice/group) for daily vehicle/RC oral gavage for 6 weeks. The four groups were vehicle, 308 (RC-1X), 615 (RC-2X), and 1,538 (RC-5X) mg/kg. The vehicle group received an equivalent volume of solution base.

### Exercise Performance Test

Exercise performance protocol was identical to our previous manuscript (23). A low-force testing system (PicoScope 2000, Pico Technology Limited, Cambridgeshire, UK) was used to measure the forelimb grip strength of mice after 5 weeks of vehicle or RC treatment and 1 h after the last treatment dose was administered. The maximal force (in grams) recorded by this low-force system was used as the grip strength. To evaluate endurance times, the swim-to-exhaustion test was conducted, with loads corresponding to 5% of mouse body weight (BW) attached to the tails.

### Fatigue-Associated Biochemical Indices, Serum Biochemical Index, and Hormone Index

Fatigue-associated biochemical indices in the serum were determined using an autoanalyzer (SYSMEX XT-2000iv, Sysmex corp., Kobe, Japan). The effects of RC on serum lactate, ammonia, creatine kinase (CK), glucose, lactate dehydrogenase (LDH), and FFA activity were evaluated postexercise. One hour after the last dose of the 41-day supplementation, a 15-min swimming test was performed without weight loading. After 1 h of the last intragastric administration of RC, in all mice serum samples were collected by cardiac puncture and analyzed using an autoanalyzer (SYSMEX XT-2000iv, Sysmex corp., Kobe, Japan). The hormones E2 and progesterone (PGN) were determined using the Roche Cobas e411 analyzer (Roche Diagnostics, Mannheim, Germany).

### Tissue Glycogen Determination and Visceral Organ Weight

Glucose is stored as glycogen, mostly in liver and muscle tissues. Liver and muscle tissues were excised after the mice were euthanized and weighed for glycogen content analysis as described previously (23). The liver, heart, lung, kidney, Ovarian fat pad (OPF), gastrocnemius muscle, and brown adipose tissue (BAT) were collected after the last intragastric administration of RC and euthanized.

### Magnetic Resonance Imaging and Histological Staining of Tissues

Magnetic resonance imaging (MRI) experiments were conducted on a 0.5T mice MRI scanner (MesoMR, Niumag analytical, Suzhou, China) after 1 h of intragastric administration of RC before anesthetizing. The mice were laid flat on a wooden board, their limbs and trunk were fixed with tape, they were placed in a nuclear magnetic resonance testing room; we selected the third cross-section for MRI imaging. Hematoxylin and eosin (H&E) staining was used for histological and pathological evaluations. After anesthetizing the mice, their tissues were fixed with 10% formalin for histopathological analysis. A clinical pathologist then stained the tissue sections with H&E and examined them under a light microscope with a CCD camera (BX-51, Olympus, Tokyo, Japan).

### Bacterial DNA Extraction and 16S rRNA Sequencing

The procedures for sample preparation and extraction were previously described (23). Microbiome bioinformatics were mainly performed with QIIME 2 (24), whereas the OTU clustering procedure was performed with the Vsearch (v2.13.4) (25) pipeline described here (https://github.com/torognes/vsearch/wiki/VSEARCH-pipeline). Briefly, raw sequence data were demultiplexed using the demux plugin followed by primer cutting with a cutadapt plugin (26). The differences in microbiota composition were analyzed using the orthogonal partial least square-discriminant analysis (OPLS-DA). After the groups were defined, a supervised the OPLS-DA model was applied to all microbiota. The Raw sequence reads project databases are available at https://www.ncbi.nlm.nih.gov/sra/PRJNA673999.

### Statistical Analysis

All data are expressed as mean ± SEM. Homogeneity of variance was examined by Levene's test, and the normality of the data was examined by the Shapiro–Wilk test. Statistical differences between groups were analyzed using one-way analysis of variance (ANOVA) was employed to calculate the significance differences between multiple groups with Duncan's test, and the Cochran–Armitage test was used for the dose–effect trend analysis. All statistical analyses were performed using SPSS version 18.0 (SPSS, Chicago, IL, USA), and *p* < 0.05 was considered statistically significant.

## Results

### Effect of 6-Week RC Supplementation on Tissue Weight, BW, Body Composition, and Diet Intake

**Figure 1:**
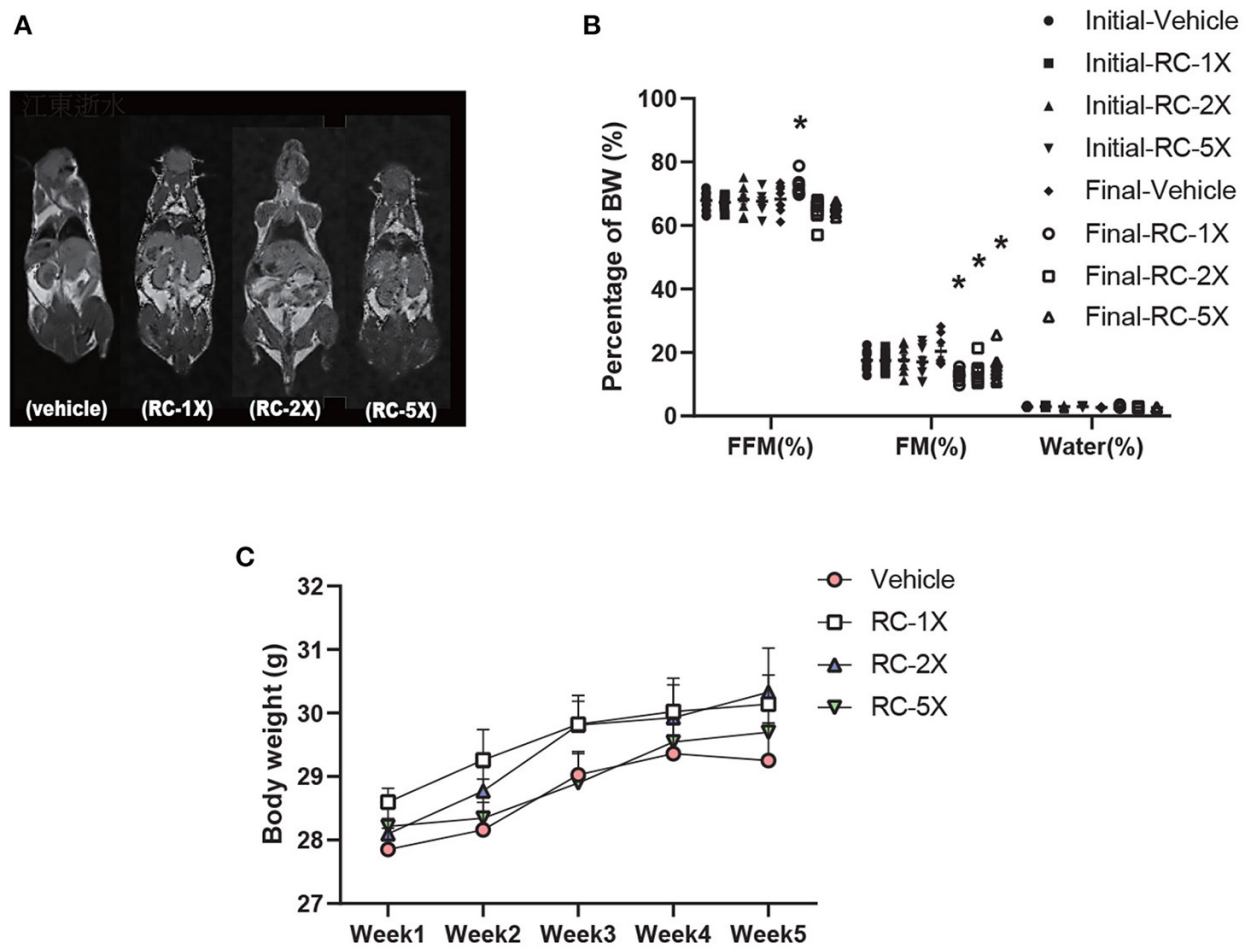
MRI examination to determine the body composition of RC-supplemented mice **(A)**; body composition can be determined based on two points. The initial test was performed before RC supplementation, and the final test was performed 6 weeks after RC supplementation **(B)**. Increased BW was observed in RC-treated mice. Female ICR mice were supplemented with vehicle, RC-1X, RC-2X, and RC-5X for 6 weeks. Values are presented as mean ± SEM for *n* = 8 mice per group **(C)**. Asterisk (*) indicates a statistically significant difference compared vehicle group (*p* < 0.05).

Table 1 shows the BW, food and water intake, and tissue weight of the mice receiving RC supplementation for 6 weeks. The initial BW, final BW, and water intake were not significantly different between the groups. However, food intake exhibited a significant increase in RC-5X mice (*p* = 0.0033) compared with the vehicle group. Tissues and organs were obtained from the sacrificed mice after the study period and weighed. No significant difference was observed in the liver, lung, kidney, heart, kidney, muscle, and brown adipose tissues of the mouse groups. Furthermore, the relative weights of all tissues were the same except brown adipose tissue (BAT). Both BAT and relative BAT weight had a dose-dependent decrease (all *p* < 0.03) by RC supplementation. MRI images, presented in Figure 1A, clearly show that the free fat mass (FFM) was predominantly located in lower bodies of mice, particularly around the genitals. Furthermore, no adverse events were observed in RC-1X, RC-2X, and RC-5X mice that received 6-week supplementation. The details of body composition data were obtained using a mice body composition analyzer procedure (mesoQMR, Niumag analytical, Suzhou, China; Figure 1B). The FFM was significantly higher in the RC-1X (1.06-fold, *p* = 0.0195) compared with the vehicle group. The fat mass (FM) was significantly lower in the RC-1X (20.38%, *p* = 0.005), RC-2X (32.53%, *p* = 0.0020), and RC-5X (23.75%, *p* = 0.0188) groups than in the vehicle group. The mice exhibited increased BW stability during the study period, and no significant difference was observed between the groups in each week (Figure 1C).

**Table 1:** Physical characteristics of mice with RC supplementation.

**Characteristic**	**Vehicle**	**RC-1X**	**RC-2X**	**RC-5X**	**Trend analysis**
Initial BW (g)	28.1 ± 0.3	28.35 ± 0.3	28.19 ± 0.2	28.13 ± 0.3	0.7120
Final BW (g)	29.56 ± 0.5	29.83 ± 0.6	30.48 ± 0.7	29.59 ± 0.4	0.8775
Food intake (g/day)	4.22 ± 0.3^a^	4.12 ± 0.2^a^	4.3 ± 0.2^a^	4.84 ± 0.4^b^	0.0074
Water intake (g/day)	7.14 ± 0.4	7.09 ± 0.5	7.31 ± 0.6	6.69 ± 0.6	0.3579
Liver (g)	1.18 ± 0.01	1.19 ± 0.05	1.19 ± 0.03	1.18 ± 0.05	0.8451
Lung (g)	0.19 ± 0.00	0.19 ± 0.01	0.20 ± 0.01	0.20 ± 0.01	0.3286
Heart (g)	0.16 ± 0.01	0.15 ± 0.01	0.16 ± 0.01	0.16 ± 0.01	0.8432
Kidney (g)	0.33 ± 0.01^a^	0.31 ± 0.01^ab^	0.32 ± 0.01^b^	0.33 ± 0.01^a^	0.6875
Muscle (g)	0.32 ± 0.01	0.32 ± 0.01	0.31 ± 0.01	0.30 ± 0.02	0.5318
BAT (g)	0.048 ± 0.01^b^	0.048 ± 0.02^b^	0.042 ± 0.01^ab^	0.041 ± 0.02^a^	<0.001
Relative liver weight (%)	4.04 ± 0.06	4.18 ± 0.09	4.11 ± 0.11	4.23 ± 0.13	0.1825
Relative lung weight (%)	0.63 ± 0.02^a^	0.71 ± 0.02^b^	0.67 ± 0.02^ab^	0.71 ± 0.03^b^	0.243
Relative heart weight (%)	0.54 ± 0.03	0.53 ± 0.03	0.55 ± 0.03	0.56 ± 0.02	0.8321
Relative kidney weight (%)	1.09 ± 0.02^abc^	1.10 ± 0.04^bd^	1.13 ± 0.03^c^	1.20 ± 0.02^d^	0.1152
Relative muscle weight (%)	1.07 ± 0.04	1.12 ± 0.04	1.10 ± 0.04	1.09 ± 0.06	0.7984
Relative BAT weight (%)	0.160 ± 0.004^a^	0.173 ± 0.006^b^	0.148 ± 0.004^a^	0.146 ± 0.007^a^	0.0325

*Data are presented as mean ± SEM, n = 8 mice/group. Different letters (a,b) in the same row indicate a significant difference at p < 0.05. Muscle mass includes both gastrocnemius and soleus muscles at the back part of the lower legs. The mice were supplemented for 6 weeks with vehicle (glucose water), RC-1X (308 mg/kg/day RC), RC-2X (615 mg/kg/day RC), or RC-5X (1,538 mg/kg/day RC). BW, body weight; BAT, brown adipose tissue*.

### Effect of 6-Week RC Supplementation on Exercise Performance and Muscle Glycogen Content

**Figure 2:**
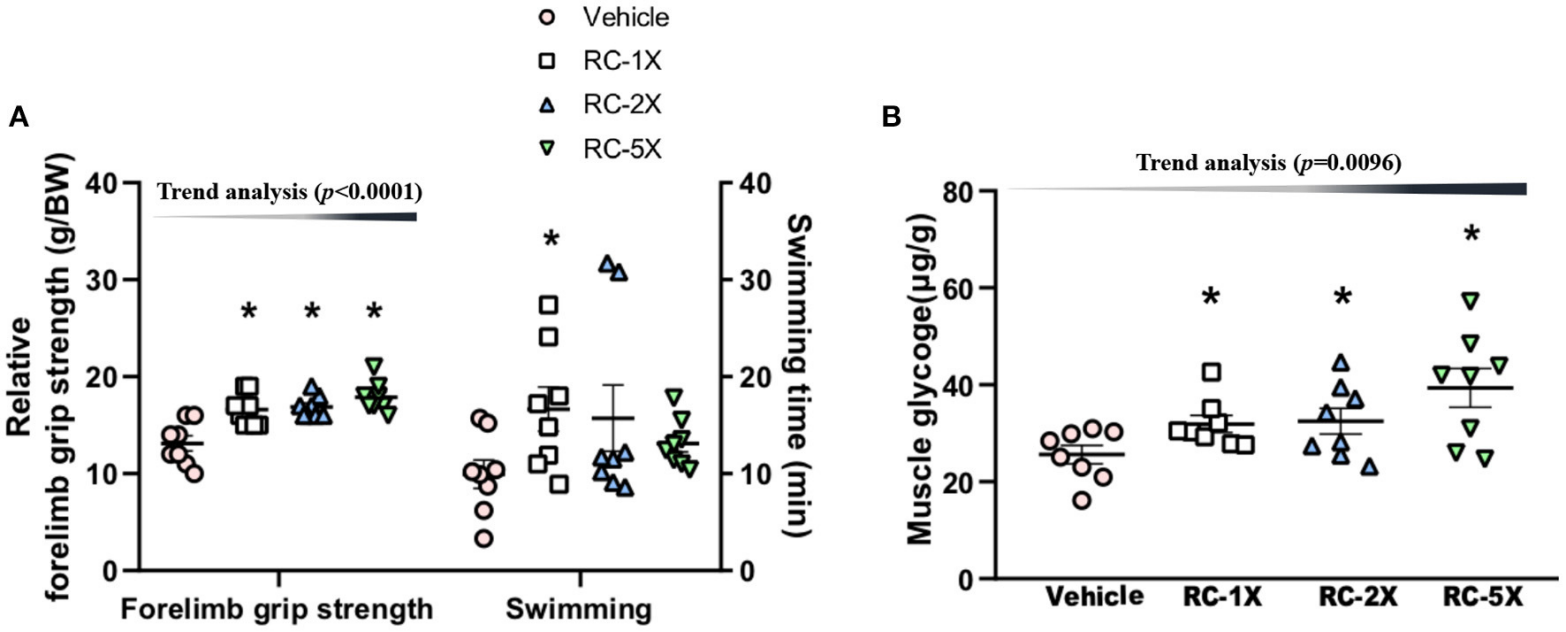
Effect of RC supplementation on exercise performance **(A)**. Female ICR mice were pretreated with vehicle, RC-1X, RC-2X, and RC-5X and underwent exercise performance test 1 h after the final administered dose. The effect of RC on hepatic glycogen levels at rest **(B)**. All mice were sacrificed and examined for glycogen levels of muscle tissues 1 h after the final treatment. Data represent the mean ± SEM of eight mice in each group. One-way ANOVA was used for the analysis. Asterisk (*) indicates a statistically significant difference compared vehicle group (*p* < 0.05).

The relative grip strength was normalized and then divided by each mouse's BW; the relative grip strength values of the vehicle, RC-1X, RC-2X, and RC-5X groups were 13.2 ± 0.8, 16.5 ± 0.6, 16.8 ± 0.5, and 17.7 ± 0.5 g/BW, respectively (Figure 2A). The relative grip strength values of the RC-1X, RC-2X, and RC-5X groups were significantly higher by 1.26 (*p* = 0.0003), 1.27 (*p* = 0.0001), and 1.35 (*p* < 0.0001) times that of the vehicle group. The exhaustive swimming times of the vehicle, RC-1X, RC-2X, and RC-5X groups were 9.9 ± 1.5, 16.7 ± 2.3, 15.7 ± 3.4, and 13.1 ± 2.5 min, respectively, with the value of the RC-1X group being 1.67-fold higher than that of the vehicle group (*p* = 0.0412). The grip strength had a significant dose-dependent increase (*p* < 0.0001) in RC supplement whereas the swimming time had no significant difference in dose-dependent effect (*p* = 0.1131).

The muscle glycogen values of the vehicle, RC-1X, RC-2X, and RC-5X groups were 25.6 ± 5.3, 31.9 ± 4.9, 32.5 ± 7.6, and 39.3 ± 11.3 μg/g, respectively (Figure 2B). The RC-1X, RC 2X, and RC-5X muscle glycogen content was significantly higher by 1.28-, 1.30-, and 1.54-fold (all *p* < 0.04) compared with the vehicle group. Furthermore, a significant dose-dependent effect on muscle glycogen content was observed (*p* = 0.0096).

### Effect of 6-Week RC Supplementation on Fatigue-Related Profile After the 15-Min Swimming Test

**Figure 3:**
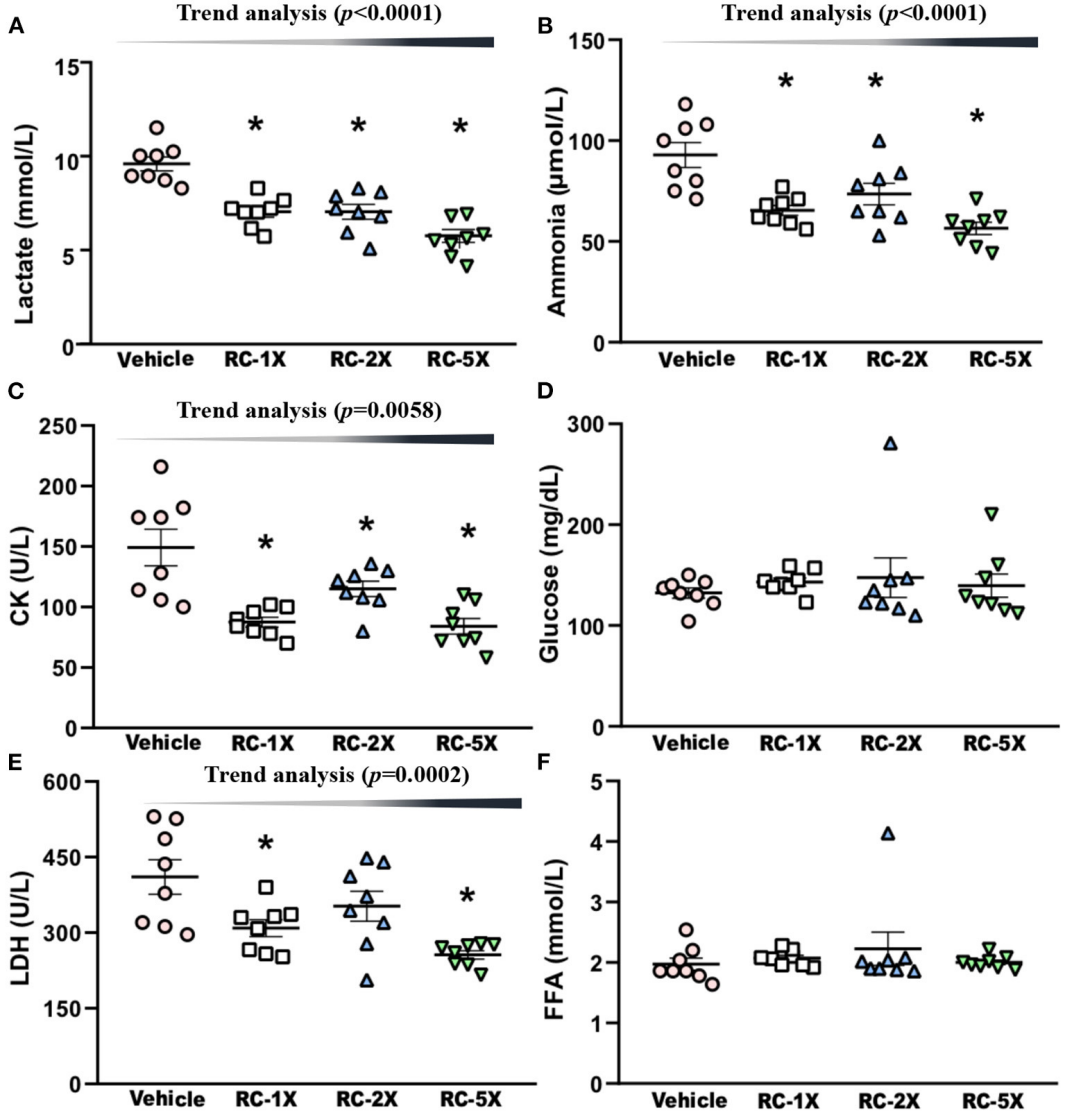
Effect of RC supplementation on acute exercise biochemical test. Lactate **(A)**; ammonia **(B)**; CK **(C)**; glucose **(D)**; LDH **(E)**; and FFA **(F)**. Female ICR mice were pretreated with the vehicle, RC-1X, RC-2X, and RC-5X and underwent exercise performance test 1 h after the final administered dose. The effect of RC on hepatic glycogen levels at rest **(B)**. All mice performed swimming exercise without loading and were examined for fatigue-related biochemical indices 1 h after the RC treatment. Data represent the mean ± SEM of eight mice in each group. One-way ANOVA was used for the analysis. Asterisk (*) indicates a statistically significant difference compared vehicle group (*p* < 0.05).

On Day 43 of the supplementation period, mice underwent a 15-min swimming test to evaluate the levels of fatigue-related biochemical index. Serum lactate levels in the vehicle, RC-1X, RC-2X, and RC-5X groups were 10.4 ± 0.3, 8.0 ± 0.3, 8.0 ± 0.4, and 6.5 ± 0.3 mmol/L, respectively, and compared with the vehicle group, the lactate levels in the other groups were significantly lower at 22.84% (*p* < 0.0001), 22.84% (*p* < 0.0001), and 37.26% (*p* < 0.0001), respectively (Figure 3A). The serum ammonia levels in the vehicle, RC-1X, RC-2X, and RC-5X groups were 93 ± 6, 65 ± 2, 74 ± 5, and 57 ± 3 mmol/L, and compared with the vehicle group, the ammonia level of the other groups were significantly lower at 29.61% (*p* = 0.0002), 20.86% (*p* = 0.0052), and 39.17% (*p* < 0.0001), respectively (Figure 3B). The serum CK levels in the vehicle, RC-1X, RC-2X, and RC-5X groups were 149 ± 15, 101 ± 4, 100 ± 6, and 84 ± 6 U/L, respectively, and compared with the vehicle group, CK levels of the other groups were significantly lower (32.16%, *p* < 0.0001; 33.33%, *p* = 0.0120; and 43.72%, *p* < 0.0001, respectively; Figure 3C). The glucose levels in the vehicle and RC groups were not significantly different (Figure 3D). The LDH levels in the RC-1X (309 ± 17 U/L) and RC-5X (256 ± 8 U/L) groups were significantly lower (24.73%, *p* = 0.0067 and 37.64%, *p* = 0.0001, respectively) than in the vehicle group (411 ± 34 U/L; Figure 3E). FFA levels in the vehicle, RC-1X, RC-2X, and RC-5X groups were 1.97 ± 0.10, 2.07 ± 0.05, 2.23 ± 0.27, and 2.00 ± 0.04 mmol/L, respectively, and were not significantly different between the groups (Figure 3F). Thus, the 6 weeks RC supplementation influenced lactate, ammonia, CK, and LDH levels to reduce the fatigue biochemical index. Furthermore, the trend analysis showed a significant dose-dependent effect on the lactate (*p* < 0.0001), ammonia (*p* < 0.0001), CK (*p* = 0.0058), and LDH (*p* = 0.0002).

### Effect of 6-Week RC Supplementation on E2 and PGN

**Figure 4:**
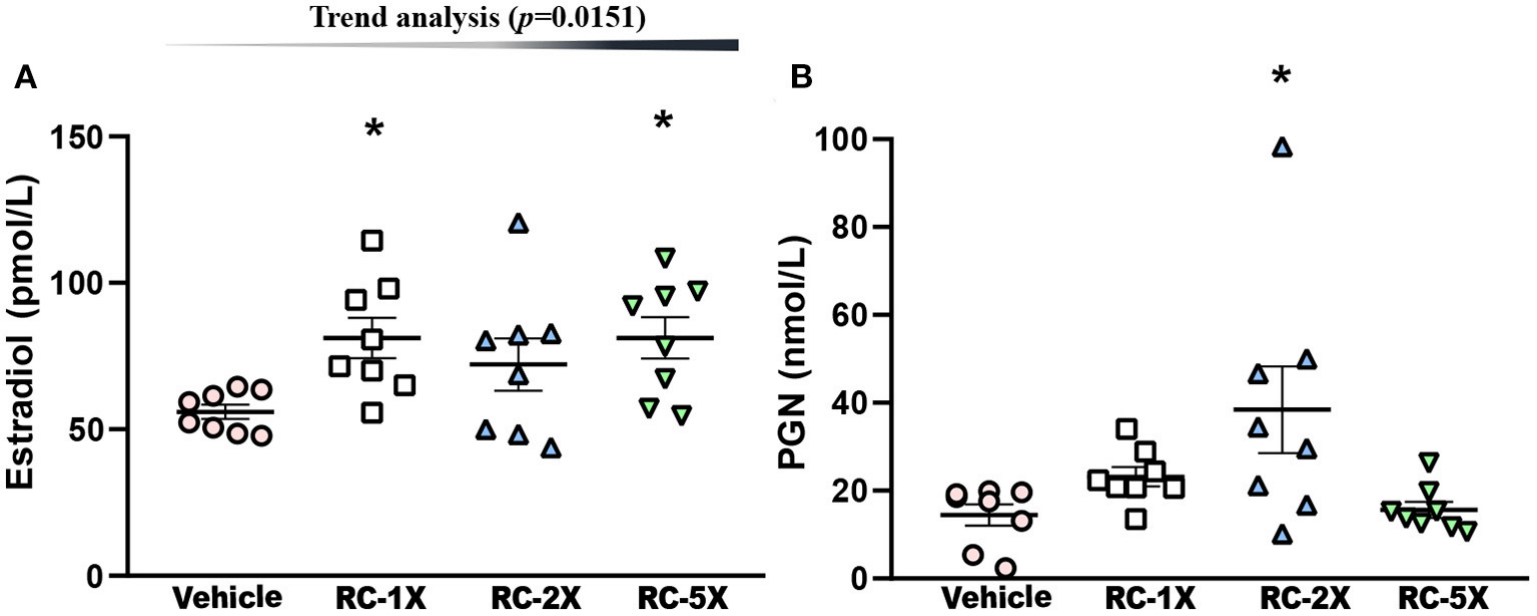
Effect of RC supplementation on **(A)** E2 and **(B)** PGN levels while mice were at rest. Mice were pretreated with the vehicle, RC-1X, RC-2X, and RC-5X for 6 weeks. All mice were sacrificed and examined for E2 and PGN levels of tissues 1 h after the final treatment. Data represent the mean ± SEM of eight mice in each group. One-way ANOVA was used for the analysis. Asterisk (*) indicates a statistically significant difference at *p* < 0.05 compared vehicle group.

Hormone indices of estrogen (E2) and PGN in regulating carbohydrate and fat use at rest and during exercise in female mice were studied (27). E2 levels in the vehicle, RC-1X, RC-2X, and RC-5X groups were 56.0 ± 2.4, 81.2 ± 6.9, 72.1 ± 9.0, and 81.2 ± 7.1 ρmol/L, and RC-1X and RC-5X had significantly higher E2 levels than the vehicle groups by 1.45- (*p* = 0.0139) and 1.45-fold (*p* = 0.0138), respectively (Figure 4A). PGN levels in the vehicle, RC-1X, RC-2X, and RC-5X groups were 14.4 ± 2.4, 23.2 ± 2.2, 38.4 ± 9.9, and 15.6 ± 1.8 nmol/L, respectively, and the PGN level in the RC-2X group was significantly higher (2.66-fold; *p* = 0.0033) than that in the vehicle group (Figure 4B). In trend analysis, the E2 have significantly dose-dependent increase (*p* = 0.0151) by RC treat. The PGN-2X have the outlier data that have no dose-dependent (*p* = 0.6250) in trend analysis.

### Effect of 6-Week RC Supplementation on Tissue Histopathology and Biochemical Variables

**Figure 5:**
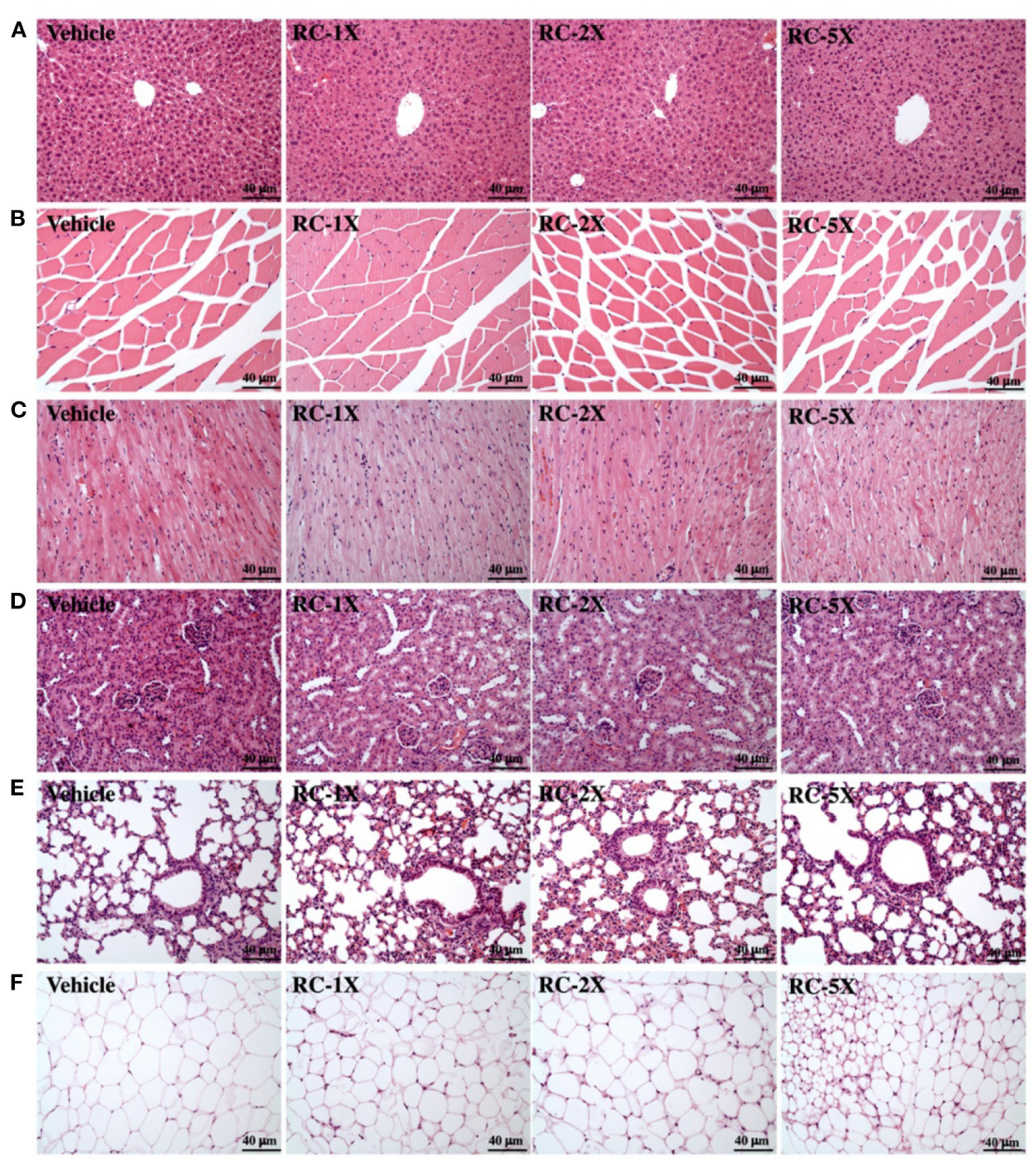
Effect of RC on the histology of various tissues: liver **(A)**; skeletal muscle **(B)**; heart **(C)**; kidney **(D)**; lungs **(E)**; and OPF **(F)**. Specimens were photographed using light microscopy. HandE stain, magnification: ×200 **(A–F)**. OPF, ovarian fat pad.

At the end of the study period, mice were sacrificed and major organs and tissues removed for histopathological analyses. Representative histopathological sections of the liver, lung, muscle, heart, kidney, and ovarian fat pad (OFP) tissues are shown in Figure 5. The results showed that no morphologic changes of sinusoid and hepatic cords in the liver of RC treatment groups compared with vehicle group. There were no hypertrophy or hyperplasia response in heart cardiomyocytes and myofibers of gastrocnemius muscles. No markedly difference in kidney of renal tubules and glomerulus with RC treatment and no alteration in the alveolar, bronchial and interstitial space in lung. The adipose tissue morphology and size of white adipocytes were not shown to be different between each group. The levels of CK and LDL in the RC group were significantly lower than those in the vehicle group. In addition, RC supplementation exhibited a dose-dependent decrease in triacyl glycerol (TG), CK, and LDL levels (Table 2).

**Table 2:** Effect of 6-week RC supplementation on biochemical variables.

**Parameter**	**Vehicle**	**RC-1X**	**RC-2X**	**RC-5X**	**Trend analysis**
AST (U/L)	112 ± 8	105 ± 8	120 ± 7	131 ± 19	0.7394
ALT (U/L)	49 ± 4	39 ± 3	46 ± 6	53 ± 9	0.7578
ALB (U/L)	37.5 ± 0.4	38.3 ± 0.4	37.6 ± 0.9	34.8 ± 3.8	0.4957
TP (G/L)	60.4 ± 1.1	64.4 ± 1	62.4 ± 1.3	57.8 ± 7.1	0.0124
UREA (mmol/L)	7.1 ± 0.2^ab^	7.1 ± 0.3^b^	6.6 ± 0.1^ab^	6.4 ± 0.4^a^	0.1619
TC (mg/dL)	140 ± 7^a^	127 ± 5^ab^	127 ± 4^b^	133 ± 5^ab^	0.2335
TG (mg/dL)	199 ± 13^ab^	202 ± 12^b^	177 ± 15^ab^	166 ± 15^a^	0.0164
GLU (mg/dL)	105 ± 6^b^	67 ± 5^a^	79 ± 5^a^	79 ± 15^b^	0.2614
CK (U/L)	1,012 ± 155^b^	412 ± 66^a^	369 ± 55^a^	246 ± 66^a^	<0.0001
LDL (mg/dL)	25.4 ± 2^b^	18.1 ± 2^a^	16.3 ± 1^a^	16.1 ± 2^a^	<0.0001
HDL (mg/dL)	116 ± 4^a^	140 ± 9^b^	138 ± 11^ab^	128 ± 8^ab^	0.2803

*Data are expressed as mean ± SEM, n = 8 mice/group. Different letters (a,b) in the same row indicate significant differences at p < 0.05 based on one-way ANOVA. AST, aspartate aminotransferase; ALT, alanine amino transferase; ALB, albumin; TP, total protein; UREA, urea; TC, total cholesterol; TG, triacyl glycerol; GLU, glucose; CK, creatine kinase; LDL, low-density lipoprotein; HDL, high-density lipoprotein cholesterol*.

### Effect of 6-Week RC Supplementation on Gut Microbiota

**Figure 6:**
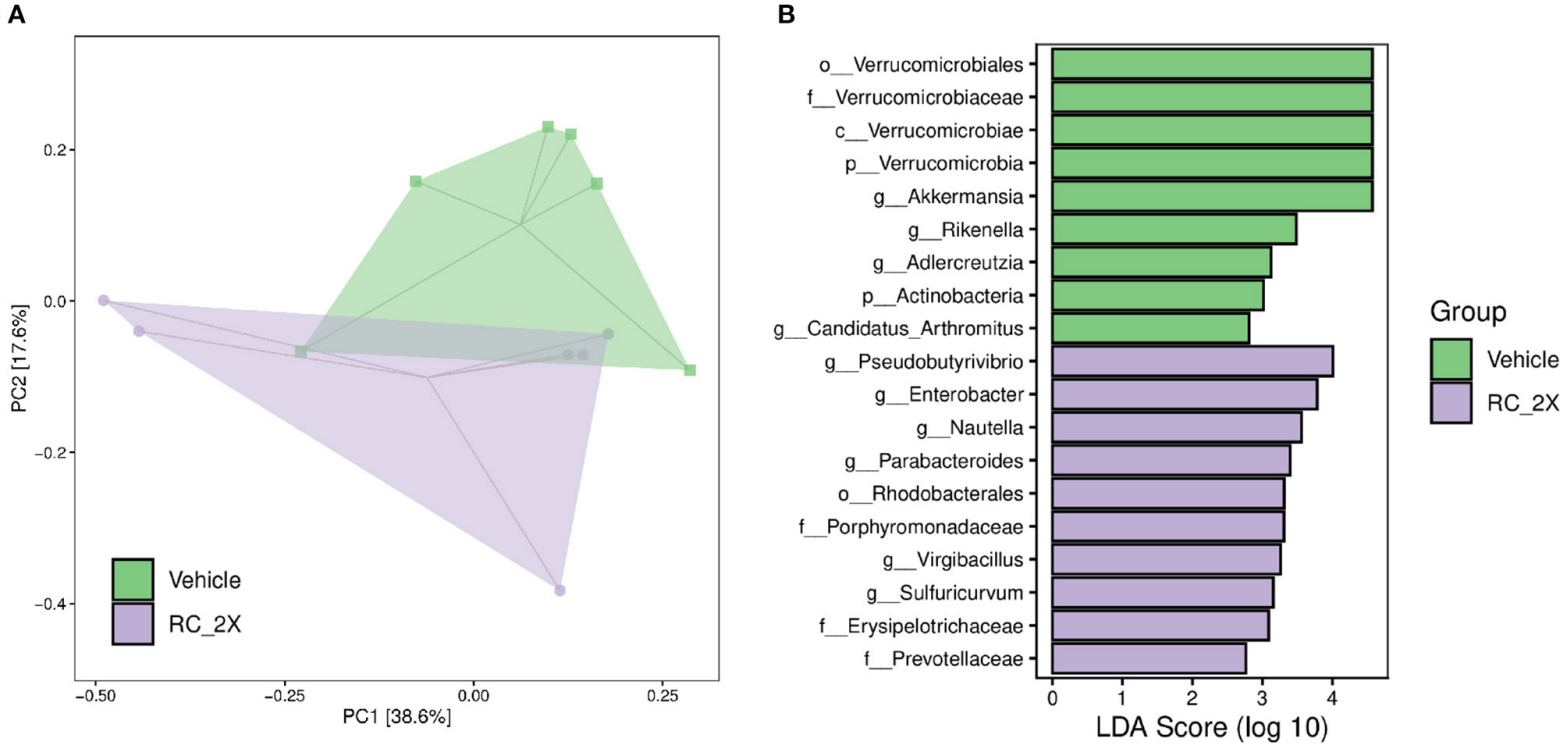
**(A)** Score plots of OPLS-DA represent differences between the vehicle and RC-2X groups based on clustering results. OPLS-DA loading plots display variables positively correlated with score plots. Statistically and significantly different separations responsible for the differentiation of the two groups were identified. **(B)** Differentially abundant bacterial groups through LDA scores based on LEfSe, and illustration showing the difference between vehicle and RC groups.

We analyzed gut microbiota composition in vehicle and RC-supplemented mice using 16S rRNA gene sequencing. Figure 6A shows the OPLS-DA. The data provide insights on differences between the vehicle and RC-2X groups. Specifically, the microbial population of the RC group was different from that of the vehicle group, suggesting that RC supplementation significantly influences the gut microbial population. Figure 6B shows that individual bacterial groups were analyzed based on LDA effect size (LEfSe). Several bacterial taxa were identified as being significantly different among the groups. The RC group exhibited an increase in the genera *Pseudobutyrivibrio, Enterobacter, Nautella, Parabacteroides, Virgibacillus*, and *Sulfuricurvum*; order *Rhodobacterales*; and families *Porphyromonadaceae, Erysipelotrichaceae*, and *Prevotellaceae*.

## Discussion

The result shows that a 6-week RC supplementation increased the FFM in mice lower bodies and decreased their FM. This is consistent with a previous study that demonstrated how isoflavone intake (75 mg/day for 12 months) significantly reduced FM in the trunk in postmenopausal (28). In addition, brown adipose tissue was significantly lowered by RC treatment. The BAT has beneficial effects on control of body composition and metabolism in 12-week-old male C57BL/6 mice (29), gut microbiota could modulate the lipid metabolism in BAT. BAT stimulates lypolysis and plays an important role in inhibiting lipogenesis in mice (30). Thus, RC supplementation could affect on lipid metabolism and have change gut microbiota. In our study, RC supplementation increased the FFM in mice lower bodies and decreased the FM. Therefore, RC increases the muscle glycogen level and thus reduces exercise fatigue and improves exercise performance. Lactate, ammonia, glucose, CK, and LDH levels are the most commonly used biomarkers for studying muscle fatigue (31, 32) in humans. A possible explanation for the observed effects is that estrogen can repress mitochondrial uncoupling and reduce muscle fatigue through increased generation of ATP molecules through mitochondrial respiration (11). The gut microbiome may play a role in reducing the accumulation of damage-induced metabolites during exercise. Previous observations indicate that the isoflavone molecular form and the intestinal microbiome active in isoflavone uptake, metabolism, and ultimately therapeutic efficacy have crucial roles in postmenopausal osteopenic women treated with 60 mg of isoflavone aglycones (33). Moreover, studies have demonstrated the potential role of RC in inhibiting chronic inflammation (34). Thus, RC supplementation may possibly relieve muscle soreness and prevent delayed-onset muscle soreness. Our results showed that 6-week RC supplementation increased estrogen and PGN levels in mice. This result may be attributed to isoflavones. Isoflavones are phytoestrogens with potent estrogenic activity (35). This may be due to estrogen receptor-α and estrogen receptor-β. Furthermore, several studies have confirmed that estrogen and PGN contribute to improving endurance exercise performance (11), muscle strength in women (15), and muscle mass (36) and reducing bone loss (3). Moreover, estrogens influence fiber atrophy, specifically fast-twitch muscle rather than slow-type muscle (37). Thus, RC contributes toward improving muscle mass and exercise performance.

In the present study, RC supplementation decreased the levels of CK, LDL, and TG in mice. Moreover, Luís et al. (8) concluded that supplementation with phytoestrogens, through their inhibition of 7a-hydroxylase and the estrogenic effects on the LDL-receptor, modulates enzyme PCSK9 to decrease LDL. This may help explain our results RC decreased the LDL. Furthermore, CK is a crucial clinical biomarker for muscle damage. TG and LDL are the main clinical biomarkers for blood lipids. The decreases in the levels of CK, LDL, and TG indicate that RC supplementation is beneficial for reducing exercise fatigue and blood lipid level. Recent studies have found RC to be effective in treating several diseases; for instance, RC is safe clinically and *in vitro* for premenopausal patients with hormone-sensitive breast cancer (38). Furthermore, a study showed that RC extract (250 mg/Kg) has a significant effect in a rat model of type 2 diabetes (39).

Flavonoids can strengthen intestinal tight junction functions and enhance barrier integrity (40). Furthermore, they can significantly upregulate the relative abundance of *Pseudobutyrivibrio* and a species of butyrate-producing bacteria present in the rumen (41). *Parabacteroides* was enhanced in the RC-2X group in a previous study; *Parabacteroide* is a gram-negative bacterium and acts as a commensal symbiont, promoting mucosal barrier health and preventing increases in several proinflammatory cytokines (42). *Virgibacillus* is gram-positive bacteria that can be isolated from human intestinal microbiota (43), and they have been found in fish sauce fermentation (44). *Virgibacillus* is a proteinase applied in functional food ingredients that has immunomodulatory activity (45). *Sulfuricurvum* is the name for sulfur-oxidizing bacteria present in the normal microflora of the skin surface of humans and mammals in general (46). The family of *Erysipelotrichaceae* was found to increase in the host with the intake of low-fat diets and hydroxypropyl methylcellulose (47). In our data, FM and TG level decreases may be correlated with *Erysipelotrichaceae* increase due to a change in lipid metabolism. Overall, RC supplementation improved intestinal barrier integrity through inducing a shift in intestinal microorganism composition. The intestinal barrier function is affected by microbiota's metabolism, whereas a decrease in barrier function during exercise might lead to changed blood flow in the gut, heart, and skeletal muscles, thus affecting exercise performance (19). In general, strenuous exercise promotes alterations in gastrointestinal permeability, thus enhancing intestinal barrier function, which reduces endotoxin release from the gut to reduce heatstroke (48). By contrast, with an increase in gastrointestinal permeability, the temperature of the core increases and blood flows away from the splanchnic area, which can directly open tight junctions and damage the enterocyte membrane, causing tissue hypoxia and oxidative stress (49). RC is an appropriate supplementation with the potential to counteract exercise-induced intestinal dysfunction by shift the positive gut microbiota.

The limitation of this study was we did not control for all female mice estrous cycle to reduce this effect, but the estrous cycle was not the main factor to alter on the exercise performance. A previous study found that estrous cycle seems to influence running economy and estrus impaired exercise heat loss, without no concomitant effect on the endurance exercise performance and grip strength in mice (50). In addition, the estrous cycle did not influence female mice muscle strength, but the estrous cycle undermined the running economy, which change the VO_2_ max in low-intensity exercise, and alter the exercise-induced thermoregulation (51). Another limitation was the fact that our intervention lasted only 6 weeks; this time frame was chosen to detect the short-term effects of RC.

Overall, RC supplementation is still an appropriate supplementation with the potential to counteract exercise-induced intestinal barrier dysfunction.

## Conclusions

In conclusion, RC supplementation improved the intestinal health by shifting microbiota composition and exerting antifatigue effects by reducing fatigue-related serum markers. According our data, RC increases forelimb grip strength and swimming time and decreases FM in female mice. RC might be useful in reducing physical fatigue, with the intestinal health contributing a critical role in physical performance, possibly enhancing the genera of *Pseudobutyrivibrio, Parabacteroide*, and *Virgibacillus* and the family *Erysipelotrichaceae*. Thus, our results show that RC is advantageous in shifting the gut microbiome and providing positive effects on female athletes' gut and overall health.

## Data Availability Statement

The original contributions presented in the study are publicly available. This data can be found here: https://www.ncbi.nlm.nih.gov/bioproject/PRJNA673999/.

## Ethics Statement

The animal study was reviewed and approved by the animal protocol (IACUC-19003) was reviewed and approved by the Institutional Animal Care and Use Committee (IACUC) of Jilin Sport University, Changchun City, China.

## Author Contributions

Y-MC and Y-SC designed the experiments. Y-MC, I-LW, and X-YZ performed the laboratory experiments. Y-MC and Y-SC analyzed the data, interpreted the results, prepared the figures, and wrote the manuscript. Y-MC, Y-SC, and W-CC contributed reagents, materials, and analysis platforms. All authors contributed to the article and approved the submitted version.

## Conflict of Interest

The authors declare that the research was conducted in the absence of any commercial or financial relationships that could be construed as a potential conflict of interest.

## Abbreviations

ICR: Institute of Cancer Research
FFA: Free fatty acid
CCD: Charge-coupled device
QIIME 2: Quantitative Insights Into Microbial Ecology 2
OUT: Operational taxonomic unit
SEM: Standard error of the mean
OPF: Ovarian fat pad
OPLS-DA: Orthogonal Projections to Latent Structures
PLS-DA: Partial least squares Discriminant Analysis
LDA: Linear discriminant analysis
LDL: low-density lipoprotein cholesterol.

## References

[1] Kolodziejczyk-Czepas J. Trifolium species-derived substances and extracts-Biological activity and prospects for medicinal applications. J Ethnopharmacol. (2012) 143:14–23. 10.1016/j.jep.2012.06.048 22771317

[2] Pisarčik M Hakl J Hrevušová Z. Effect of *Pythium oligandrum* and poly-beta-hydroxy butyric acid application on root growth, forage yield and root diseases of red clover under field conditions. Crop Prot. (2020) 127:104968. 10.1016/j.cropro.2019.104968

[3] Occhiuto F Pasquale RD Guglielmo G Palumbo D Zangla G Samperi S. Effects of phytoestrogenic isoflavones from red clover (*Trifolium pratense* L) on experimental osteoporosis. Phytother Res. (2007) 21:130–4. 10.1002/ptr.2037 17117453

[4] Dornstauder E Jisa E Unterrieder I Krenn L Kubelka W Jungbauer A. Estrogenic activity of two standardized red clover extracts (Menoflavon®) intended for large scale use in hormone replacement therapy. J Steroid Biochem Mol Biol. (2001) 78:67–75. 10.1016/S0960-0760(01)00075-9 11530286

[5] Beck V Rohr U Jungbauer A. Phytoestrogens derived from red clover: an alternative to estrogen replacement therapy? J Steroid Biochem Mol Biol. (2005) 94:499–518. 10.1016/j.jsbmb.2004.12.038 15876415

[6] Ghazanfarpour M Sadeghi R Roudsari RL Khorsand I Khadivzadeh T Muoio B. Red clover for treatment of hot flashes and menopausal symptoms: a systematic review and meta-analysis. J Obstet Gynaecol. (2016) 36:301–11. 10.3109/01443615.2015.1049249 26471215

[7] Kang SJ Choi BR Kim SH Yi HY Park HR Kim DC. Dried pomegranate potentiates anti-osteoporotic and anti-obesity activities of red clover dry extracts in ovariectomized rats. Nutrients. (2015) 7:2622–47. 10.3390/nu7042622 25912038 PMC4425164

[8] Luís Â Domingues F Pereira L. Effects of red clover on perimenopausal and postmenopausal women's blood lipid profile: a meta-analysis. Climacteric. (2018) 21:446–53. 10.1080/13697137.2018.1501673 30269660

[9] Yokoyama S-i Kodera M Hirai A Nakada M Ueno Y Osawa T. Red clover (*Trifolium pratense* L.) sprout prevents metabolic syndrome. J Nutr Sci Vitaminol. (2020) 66:48–53. 10.3177/jnsv.66.48 32115453

[10] Zhang H Zhao J Shang H Guo Y Chen S. Extraction, purification, hypoglycemic and antioxidant activities of red clover (*Trifolium pratense* L.) polysaccharides. Int J Biol Macromol. (2020) 148:750–60. 10.1016/j.ijbiomac.2020.01.194 31978472

[11] Nagai S Ikeda K Horie-Inoue K Shiba S Nagasawa S Takeda S. Estrogen modulates exercise endurance along with mitochondrial uncoupling protein 3 downregulation in skeletal muscle of female mice. Biochem Biophys Res Commun. (2016) 480:758–64. 10.1016/j.bbrc.2016.10.129 27983991

[12] Smith GI Yoshino J Reeds DN Bradley D Burrows RE Heisey HD. Testosterone and progesterone, but not estradiol, stimulate muscle protein synthesis in postmenopausal women. J Clin Endocrinol Metab. (2014) 99:256–65. 10.1210/jc.2013-2835 24203065 PMC3879672

[13] Enns DL Tiidus PM. The influence of estrogen on skeletal muscle. Sports Med. (2010) 40:41–58. 10.2165/11319760-000000000-00000 20020786

[14] Velders M Schleipen B Fritzemeier KH Zierau O Diel P. Selective estrogen receptor-β activation stimulates skeletal muscle growth and regeneration. FASEB J. (2012) 26:1909–20. 10.1096/fj.11-194779 22278942

[15] Lowe DA Baltgalvis KA Greising SM. Mechanisms behind estrogens' beneficial effect on muscle strength in females. Exerc Sport Sci Rev. (2010) 38:61. 10.1097/JES.0b013e3181d496bc 20335737 PMC2873087

[16] Chilibeck PD Cornish SM. Effect of estrogenic compounds (estrogen or phytoestrogens) combined with exercise on bone and muscle mass in older individuals. Appl Physiol Nutr Metab. (2008) 33:200–12. 10.1139/H07-140 18347673

[17] Otte JA Oostveen E Geelkerken RH Groeneveld AJ Kolkman JJ. Exercise induces gastric ischemia in healthy volunteers: a tonometry study. J Appl Physiol. (2001) 91:866–71. 10.1152/jappl.2001.91.2.866 11457804

[18] Van Wijck K Lenaerts K Van Loon LJ Peters WH Buurman WA Dejong CH. Exercise-induced splanchnic hypoperfusion results in gut dysfunction in healthy men. PLoS One. (2011) 6:e22366. 10.1371/journal.pone.0022366 21811592 PMC3141050

[19] Lamprecht M Frauwallner A. Exercise, intestinal barrier dysfunction and probiotic supplementation. Med Sport Sci. (2012) 59:47–56. 10.1159/000342169 23075554

[20] Lamprecht M Bogner S Schippinger G Steinbauer K Fankhauser F Hallstroem S. Probiotic supplementation affects markers of intestinal barrier, oxidation, and inflammation in trained men; a randomized, double-blinded, placebo-controlled trial. J Int Soc Sports Nutr. (2012) 9:45. 10.1186/1550-2783-9-45 22992437 PMC3465223

[21] Oxfeldt M Dalgaard LB Risikesan J Johansen FT Hansen M. Influence of fermented red clover extract on skeletal muscle in early postmenopausal women: a double-blinded cross-over study. Nutrients. (2020) 12:3587. 10.3390/nu12113587 33238442 PMC7700192

[22] Aubertin-Leheudre M Lord C Khalil A Dionne IJ. Six months of isoflavone supplement increases fat-free mass in obese-sarcopenic postmenopausal women: a randomized double-blind controlled trial. Eur J Clin Nutr. (2007) 61:1442–4. 10.1038/sj.ejcn.1602695 17311051

[23] Chen YM Chiu WC Chiu YS Li T Sung HC Hsiao CY. Supplementation of nano-bubble curcumin extract improves gut microbiota composition and exercise performance in mice. Food Funct. (2020) 11:3574–84. 10.1039/C9FO02487E 32271330

[24] Bolyen E Rideout JR Dillon MR Bokulich NA Abnet CC Al-Ghalith GA. Author Correction: Reproducible, interactive, scalable and extensible microbiome data science using QIIME 2. Nat Biotechnol. 37:1091. 10.1038/s41587-019-0252-6 31341288 PMC7015180

[25] Rognes T Flouri T Nichols B Quince C Mahe F. VSEARCH: a versatile open source tool for metagenomics. PeerJ. (2016) 4:e2584. 10.7717/peerj.2584 27781170 PMC5075697

[26] Martin M Rahmann S. From cutadapt to sequencetools (sqt): a versatile toolset for sequencing projects. EMBnet J. (2011) 17:35. 10.14806/ej.17.B.272

[27] D'eon T Braun B. The roles of estrogen and progesterone in regulating carbohydrate and fat utilization at rest and during exercise. J Womens Health Gend Based Med. (2002) 11:225–37. 10.1089/152460902753668439 11988133

[28] Wu J Oka J Tabata I Higuchi M Toda T Fuku N. Effects of isoflavone and exercise on BMD and fat mass in postmenopausal Japanese women: a 1-year randomized placebo-controlled trial. J Bone Miner Res. (2006) 21:780–9. 10.1359/jbmr.060208 16734394

[29] Stanford KI Middelbeek RJ Townsend KL An D Nygaard EB Hitchcox KM. Brown adipose tissue regulates glucose homeostasis and insulin sensitivity. J Clin Invest. (2013) 123:215–23. 10.1172/JCI62308 23221344 PMC3533266

[30] Mestdagh R Dumas ME Rezzi S Kochhar S Holmes E Claus SP. Gut microbiota modulate the metabolism of brown adipose tissue in mice. J Proteome Res. (2012) 11:620–30. 10.1021/pr200938v 22053906

[31] Strojnik V Komi PV. Fatigue after submaximal intensive stretch-shortening cycle exercise. Med Sci Sports Exerc. (2000) 32:1314–9. 10.1097/00005768-200007000-00020 10912899

[32] Izquierdo M Gonzalez-Izal M Navarro-Amezqueta I Calbet JA Ibanez J Malanda A. Effects of strength training on muscle fatigue mapping from surface EMG and blood metabolites. Med Sci Sport Exer. (2011) 43:303–11. 10.1249/MSS.0b013e3181edfa96 20581711

[33] Lambert MNT Thybo CB Lykkeboe S Rasmussen LM Frette X Christensen LP. Combined bioavailable isoflavones and probiotics improve bone status and estrogen metabolism in postmenopausal osteopenic women: a randomized controlled trial. Am J Clin Nutr. (2017) 106:909–20. 10.3945/ajcn.117.153353 28768651

[34] Krenn L Paper D. Inhibition of angiogenesis and inflammation by an extract of red clover (*Trifolium pratense* L.). Phytomedicine. (2009) 16:1083–8. 10.1016/j.phymed.2009.05.017 19665361

[35] Vitale DC Piazza C Melilli B Drago F Salomone S. Isoflavones: estrogenic activity, biological effect and bioavailability. Eur J Drug Metab Pharmacokinet. (2013) 38:15–25. 10.1007/s13318-012-0112-y 23161396

[36] Messier V Rabasa-Lhoret R Barbat-Artigas S Elisha B Karelis AD Aubertin-Leheudre M. Menopause and sarcopenia: a potential role for sex hormones. Maturitas. (2011) 68:331–6. 10.1016/j.maturitas.2011.01.014 21353405

[37] Kitajima Y Ono Y. Estrogens maintain skeletal muscle and satellite cell functions. J Endocrinol. (2016) 229:267–75. 10.1530/JOE-15-0476 27048232

[38] Ferraris C Ballestra B Listorti C Cappelletti V Reduzzi C Scaperrotta GP. Red clover and lifestyle changes to contrast menopausal symptoms in premenopausal patients with hormone-sensitive breast cancer receiving tamoxifen. Breast Cancer Res Treat. (2020) 180:157–65. 10.1007/s10549-020-05534-4 31975316

[39] Oza MJ Kulkarni YA. Trifolium pratense (Red Clover) Improve SIRT1 expression and glycogen content in high fat diet-streptozotocin induced type 2 diabetes in rats. Chem Biodivers. (2020) 17:e2000019. 10.1002/cbdv.202000019 32187456

[40] Suzuki T Hara H. Role of flavonoids in intestinal tight junction regulation. J Nutr Biochem. (2011) 22:401–8. 10.1016/j.jnutbio.2010.08.001 21167699

[41] de Paiva CS Jones DB Stern ME Bian F Moore QL Corbiere S. Altered mucosal microbiome diversity and disease severity in sjogren syndrome. Sci Rep. (2016) 6:23561. 10.1038/srep23561 27087247 PMC4834578

[42] Arnolds KL Lozupone CA. Striking a balance with help from our little friends - How the gut microbiota contributes to immune homeostasis. Yale J Biol Med. (2016) 89:389–95. 27698623 PMC5045148

[43] Seck E Rathored J Khelaifia S Croce O Robert C Couderc C. *Virgibacillus senegalensis* sp. nov, a new moderately halophilic bacterium isolated from human gut. New Microbes New Infect. (2015) 8:116–26. 10.1016/j.nmni.2015.09.014 26693281 PMC4660226

[44] Montriwong A Kaewphuak S Rodtong S Roytrakul S Yongsawatdigul J. Novel fibrinolytic enzymes from *Virgibacillus halodenitrificans* SK1-3-7 isolated from fish sauce fermentation. Process Biochem. (2012) 47:2379–87. 10.1016/j.procbio.2012.09.020

[45] Toopcham T Mes JJ Wichers HJ Yongsawatdigul J. Immunomodulatory activity of protein hydrolysates derived from *Virgibacillus halodenitrificans* SK1-3-7 proteinase. Food Chem. (2017) 224:320–8. 10.1016/j.foodchem.2016.12.041 28159274

[46] Handley KM Bartels D O'Loughlin EJ Williams KH Trimble WL Skinner K. The complete genome sequence for putative H2- and S-oxidizer *Candidatus sulfuricurvum* sp., assembled *de novo* from an aquifer-derived metagenome. Environ Microbiol. (2014) 16:3443–62. 10.1111/1462-2920.12453 24628880

[47] Cox LM Cho I Young SA Anderson WH Waters BJ Hung SC. The nonfermentable dietary fiber hydroxypropyl methylcellulose modulates intestinal microbiota. FASEB J. (2013) 27:692–702. 10.1096/fj.12-219477 23154883 PMC3545536

[48] Hales JR. Hyperthermia and heat illness. Pathophysiological implications for avoidance and treatment. Ann NY Acad Sci. (1997) 813:534–44. 10.1111/j.1749-6632.1997.tb51743.x 9100931

[49] Vargas N Marino F. Heat stress, gastrointestinal permeability and interleukin-6 signaling - Implications for exercise performance and fatigue. Temperature. (2016) 3:240–51. 10.1080/23328940.2016.1179380 27857954 PMC4964994

[50] Aguiar ASJr Speck AE Amaral IM Canas PM Cunha RA. The exercise sex gap and the impact of the estrous cycle on exercise performance in mice. Sci Rep. (2018) 8:10742. 10.1038/s41598-018-29050-0 30013130 PMC6048134

[51] Brager AJ Heemstra L Bhambra R Ehlen JC Esser KA Paul KN. Homeostatic effects of exercise and sleep on metabolic processes in mice with an overexpressed skeletal muscle clock. Biochimie. (2017) 132:161–5. 10.1016/j.biochi.2016.11.014 27916643 PMC5191931

