# Supplementary material for: Red Clover Isoflavones Influence Estradiol Concentration, Exercise Performance, and Gut Microbiota in Female Mice
Source: Front Nutr. 2021 Apr 14;8:623698. doi: 10.3389/fnut.2021.623698 (PMC8079722; doi:10.3389/fnut.2021.623698)
Supplement: Supplementary file 1 [file Data_Sheet_1.pdf]

Table S1. Before 6-week RC supplementation on female mice exercise performance

**Exercise type**	**Vehicle**	**RC-1X**	**RC-2X**	**RC-5X**	***P*-Value**
Swimming time (min)	6.44 ± 0.60	5.61 ± 0.82	5.56 ± 0.77	5.25 ± 0.86	0.7305
Grip strength (g/g)	17.34 ± 1.11	17.19 ± 0.93	17.52 ± 0.85	16.98 ± 1.23	0.9859
Running time (min)	8.64 ± 1.25	9.63 ± 1.06	8.98 ± 1.65	9.49 ± 1.70	0.9589

Data are expressed as mean ± SEM, n = 8 mice/group. Different letters (a, b) in the same row indicate significant differences at $p < .05$ based on one-way ANOVA.
